# Modified STOP-Bang for predicting perioperative adverse events in the Thai population

**DOI:** 10.1186/s12871-021-01347-0

**Published:** 2021-04-27

**Authors:** Lisa Sangkum, Chama Wathanavaha, Visasiri Tantrakul, Munthana Pothong, Cherdkiat Karnjanarachata

**Affiliations:** 1 270 Department of Anesthesiology, Faculty of Medicine, Ramathibodi hospital, Mahidol University, Rama VI road, Phayathai, Ratchatewi, Bangkok, 10400 Thailand; 2 270 Sleep Disorder Center and Division of Pulmonary and Critical Care, Medicine Department, Ramathibodi hospital, Mahidol University, Rama VI road, Phayathai, Ratchatewi, Bangkok, 10400 Thailand

**Keywords:** Obstructive sleep apnea, Perioperative complications, Postoperative complications, Difficult airway, STOP-Bang questionnaire

## Abstract

**Background:**

Undiagnosed obstructive sleep apnea (OSA) is associated with adverse perioperative outcomes. The STOP-Bang questionnaire is a validated screening tool for OSA. However, its precision may vary among different populations. This study determined the association between high-risk OSA based on the modified STOP-Bang questionnaire and perioperative adverse events.

**Methods:**

This cross-sectional study included patients undergoing elective surgery from December 2018 to February 2019. The modified STOP-Bang questionnaire includes a history of Snoring, daytime Tiredness, Observed apnea, high blood Pressure, Body mass index > 30 kg/m^2^, Age > 50, Neck circumference > 40 cm, and male Gender. High risk for OSA was considered as a score ≥ 3.

**Results:**

Overall, 400 patients were included, and 18.3% of patients experienced perioperative adverse events. On the basis of modified STOP-Bang, the incidence of perioperative adverse events was 23.2 and 13.8% in patients with high risk and low risk (*P*-value 0.016) (Original STOP-Bang: high risk 22.5% vs. low risk 14.7%, P-value 0.043). Neither modified nor original STOP-Bang was associated with perioperative adverse events (adjusted OR 1.91 (95% CI 0.99–3.66), P-value 0.055) vs. 1.69 (95%CI, 0.89–3.21), P-value 0.106). Modified STOP-Bang ≥3 could predict the incidence of difficult ventilation, laryngoscopic view ≥3, need for oxygen therapy during discharge from postanesthetic care unit and ICU admission.

**Conclusions:**

Neither modified nor original STOP-Bang was significantly associated with perioperative adverse events. However, a modified STOP-Bang ≥3 can help identify patients at risk of difficult airway, need for oxygen therapy, and ICU admission.

**Trial registrations:**

This study was registered on Thai Clinical Trials Registry, identifier TCTR20181129001, registered 23 November 2018 (Prospectively registered).

**Supplementary Information:**

The online version contains supplementary material available at 10.1186/s12871-021-01347-0.

## Background

Obstructive sleep apnea (OSA) is a common breathing disorder in patients undergoing surgery, but it remains largely undiagnosed [1]. OSA is characterized by repetitive episodes of partial and complete upper airway obstruction, causing a reduction of airflow during sleep and leading to multiple intermediate mechanisms, such as intermittent hypoxia, high sympathetic nervous activity, endothelial dysfunction, and oxidative stress [2]. These effects may lead to neurocognitive and cardiovascular sequelae [3].

The cardiorespiratory consequences of OSA may be exacerbated in the perioperative setting because of the adverse effects of anesthetics or analgesics [4]. Therefore, it is crucial to identify high-risk of OSA in patients undergoing surgery. The gold standard for the diagnosis of OSA is the polysomnogram, which is time consuming, labor intensive, and costly. Therefore, OSA remains undiagnosed in most preoperative patients, thus increasing the risk of negative postoperative adverse outcomes, such as cardiac events (arrhythmia and myocardial infarction), pulmonary complications (reintubation, atelectasis, pneumonia, and respiratory failure), rate of unplanned intensive care unit (ICU) admission, and prolonged hospital stay [5].

Recently, the STOP-Bang questionnaire (Snoring, daytime Tiredness, Observed apnea, high blood Pressure, Body mass index [BMI] > 35 kg/m^2^, Age > 50, Neck circumference > 40 cm, male Gender) was validated as a screening modality for OSA in the preoperative setting [6].

Seet et al. [7] indicated that the STOP-Bang questionnaire may predict intraoperative and early postoperative adverse events, including hypoxia, broncho/laryngospasm, arrhythmia, hyper/hypotension, and unplanned ICU admission; the risk of these events is nearly 3.4 times higher than patients identified as high risk than those identified as low risk with the STOP-Bang questionnaire. A meta-analysis revealed that a high STOP-Bang score was associated with nearly four-fold increased odds of cardiorespiratory complications [8]. However, some studies have shown inconsistent results [9, 10].

Clinical studies using the STOP-Bang questionnaire have been conducted mainly in North American and European regions. Important sleep apnea risk factors, such as ethnicity and body mass index, together with anthropometric factors considerably influence disease prediction. Moreover, the prevalence of obesity varies among different regions [11]. Li et al. performed a cephalometric analysis in Asian and white men diagnosed with OSA. At the same degree of respiratory disturbance index, age, and body mass index, Asian men were found to be nonobese (mean BMI 26.7 ± 3.8) and had smaller cranial base dimensions [12]. Therefore, some parameters in the original STOP-Bang questionnaire may not apply to the Asian population. This hypothesis was confirmed by Banhiran et al. [13], who demonstrated greater diagnostic properties among the Thai population by using a BMI cutoff of > 30 kg/m^2^ rather than > 35 kg/m^2^. Ong et al. confirmed this by assessing the validity of the cutoff used to score BMI in the questionnaire among Asian patients [14]. Because screening tests are required to have highly sensitive performance, our study used a BMI cutoff of 30 kg/m^2^. We hypothesized that the modified STOP-Bang would have a greater association with perioperative adverse outcomes than the original STOP-Bang questionnaire.

## Methods

### Patient population

In this cross-sectional study, we included adult patients (> 18 years of age) with American Society of Anesthesiologists (ASA) class I–III who were scheduled to undergo non-cardiothoracic surgery from December 2018 to February 2019. All patients provided written informed consent before participating in the study, and the study was performed in accordance with the Declaration of Helsinki. Patients were excluded if they had an artificial airway (e.g., endotracheal tube, tracheostomy), a history of poorly controlled pulmonary disease, and undergoing bariatric surgery.

The study was approved by the ethics committee of Ramathibodi Hospital, Mahidol University, Bangkok, Thailand (ID 04–61-51) and was registered on the Thai Clinical Trials Registry, identifier TCTR20181129001, registered 23 November 2018 (Prospectively registered) http://www.clinicaltrials.in.th/index.php?tp=regtrials&menu=trialsearch&smenu=fulltext&task=search&task2=view1&id=4205. The reporting of this study was performed by adhering to the Standards for Reporting of Diagnostic Accuracy Studies (STARD) statement for the reporting of diagnostic accuracy studies.

Our primary objective was to investigate the association between the incidence of perioperative adverse events and OSA risk (high vs. low) based on the original and modified STOP-Bang questionnaires. Our secondary objective was to identify the incidence of difficult facemask ventilation, difficult intubation, Cormack–Lehane classification of laryngoscopic view ≥3, need for oxygen supplement during the recovery period, intensive care unit admission, risk factors of perioperative adverse events, and 30-day mortality.

### Instruments and definitions

The modified STOP-Bang questionnaire was used by anesthesiologists during the preoperative period. The investigators determined the occurrence of postoperative complications (cardiovascular, pulmonary), difficult ventilation, difficult intubation, Cormack–Lehane classification of laryngoscopic view ≥3 and unexpected ICU admission rates.

The modified STOP-Bang includes eight items: snoring, tiredness, observe apnea, high blood pressure, BMI > 30 kg/m^2^, age > 50 years, neck circumference > 40 cm, and male gender. Patients who had STOP-BANG scores ≥3 identified as high risk for OSA.

Difficult ventilation was defined as facemask ventilation that is not adequately ventilated after inserting the oral airway or required using a two-person technique.

Difficult intubation was defined as ≥3 attempts of intubation by an experienced anesthesiologist or an intubation period of > 10 min.

The perioperative adverse events included a history of hypoxemia, reintubation, arrhythmia, myocardial infarction, congestive heart failure, respiratory adverse events and hypertensive events, which were reported by the attending anesthetist. The definitions are detailed in Additional file 1.

### Data analysis and statistics

#### Sample size estimation

The sample size calculation was based on the prevalence of postoperative complications among patients with high-risk OSA based on the original STOP-Bang questionnaire [15], which was 19.6%. The confidence level (1 − α) was used for statistical reporting, where α = 0.05 and precision (e) was 0.04. The required sample size was 385 participants.

#### Data analysis

Statistical analyses were performed using SPSS v 22.0 (SPSS, Chicago, IL, USA). Data on patient characteristics are presented as mean ± standard deviation or frequency (percentage). The relationship between risk of OSA, based on a modified STOP-Bang questionnaire, and perioperative complications was evaluated using the chi-square test. For pairwise relationships, a two-sample t test was used to compare continuous variables and the chi-square test for binary variables.

Performance predictors, including sensitivity, specificity, positive likelihood ratio (+LR), and negative likelihood ratio (−LR), were calculated and compared. The receiver operating characteristic curve (ROC) was used to compare the ability of the original and modified STOP-Bang questionnaires to predict perioperative adverse events.

Logistic regression was used to determine the association between OSA risk and perioperative adverse events. Covariates included ASA physical status, preoperative oxygen saturation, type of anesthesia, and opioid dosage, were categorized to account for the potential association of adverse outcomes with each predictor. *P* <  0.05 was set as significant.

## Results

**Fig. 1:**
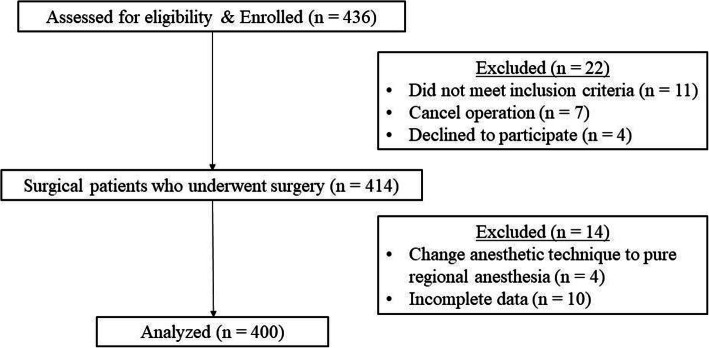
Flow diagram illustrating patient enrolment and final analysis

Four hundred patients met the inclusion criteria, completed the questionnaire, and were included in the analysis (Fig. 1). The baseline demographic data are summarized in Table 1. On the basis of the modified STOP-Bang questionnaire, 190 and 210 patients were identified as high and low risk for OSA, respectively. Compared with the low-risk group, the high-risk group was older; had higher body weight; had a higher proportion of male patients; and had a higher incidence of diabetes, hypertension, and dyslipidemia. Most patients received general anesthesia. Exploratory laparotomy, laparoscopic surgery, and superficial surgery (e.g., breast surgery or hernia repair) were the main surgical procedures performed in the study patients.

**Table 1:** Patient characteristics and demographics identify risk of OSA based on modified STOP-Bang questionnaire

Variable	Low risk for OSA (***n*** = 210)	High risk for OSA (***n*** = 190)	***P***-value
Age (years), mean ± SD	47.3 ± 13.80	61.1 ± 12.6	< 0.001
Sex, n (%)			< 0.001
• Male	31 (14.8%)	100 (52.6%)	
• Female	179 (85.2%)	90 (47.4%)	
BMI (kg/m2), mean ± SD	23.2 ± 3.6	26.8 ± 5.1	< 0.001
Preoperative oxygen saturation (%)	98.7 ± 1.6	98.0 ± 2.0	0.001
Underlying disease, n (%)			
• Diabetes mellitus	20 (9.5%)	51 (26.8%)	< 0.001
• Hypertension	41 (19.5%)	131 (68.9%)	< 0.001
• Dyslipidemia	35 (16.7%)	54 (28.6%)	< 0.001
• Coronary disease	1 (0.5%)	5 (2.6%)	0.077
• History of congestive heart failure	0 (0%)	1 (0.5%)	0.293
• History of arrythmia	4 (1.9%)	6 (3.2%)	0.423
• Pulmonary disease	5 (2.4%)	6 (3.2%)	0.72
• Renal disease	21 (10%)	24 (12.7%)	0.74
Operation type, n (%)			
• Explore laparotomy	68 (32.4%)	46 (24.1%)	0.025
• Orthopedic surgery	37 (17.5%)	42 (22.3%)	
• Laparoscopic surgery and endoscopic surgery	43 (20.3%)	46 (24.5%)	
• Breast surgery, Excision or Repair hernia	44 (20.7%)	27 (19.9%)	
• Others	20 (9.4%)	17 (9.0%)	
Anesthetic technique and operation time			
• GA, n (%)	132 (69.5%)	134 (63.8%)	0.032
• GA combined regional anesthesia, n (%)	18 (8.6%)	28 (14.7%)	0.054
o GA + Interscalene brachial plexus block	12	15	
o GA + Epidural analgesia	6	13	
• Spinal anesthesia, n (%)	40 (19.1%)	48 (25.3%)	0.134
• Operation Time, median (IQR)	160 (110–210)	165 (105–230)	0.646
Total fluid & Estimated blood loss			
• Estimate blood loss (ml), median (IQR)	100 (20–250)	50 (20–300)	0.022
• Total IV fluid (ml), median (IQR)	1100 (650–1850)	1010 (700–2000)	0.463
• Blood component, n (%)	10 (4.8%)	8 (4.2%)	0.791
Post-operative outcomes			
• Post-operative ICU admission, n (%)	5 (2.4%)	15 (7.9%)	0.007
• Post-operative unplanned ICU admission, n (%)	0	3 (1.6%)	0.106
• 30-days mortality, n (%)	0	0	N/A
• Length of hospital stay (days), median (IQR)	4 (3, 5)	4 (3, 6)	1.00

*GA* General anesthesia, *IQR* Interquartile range, *IV* Intravenous fluid, *Kg* Kilogram, *ML* Milliliter, *OSA* Obstructive sleep apnea, *SD* Standard deviation

**Fig. 2:**
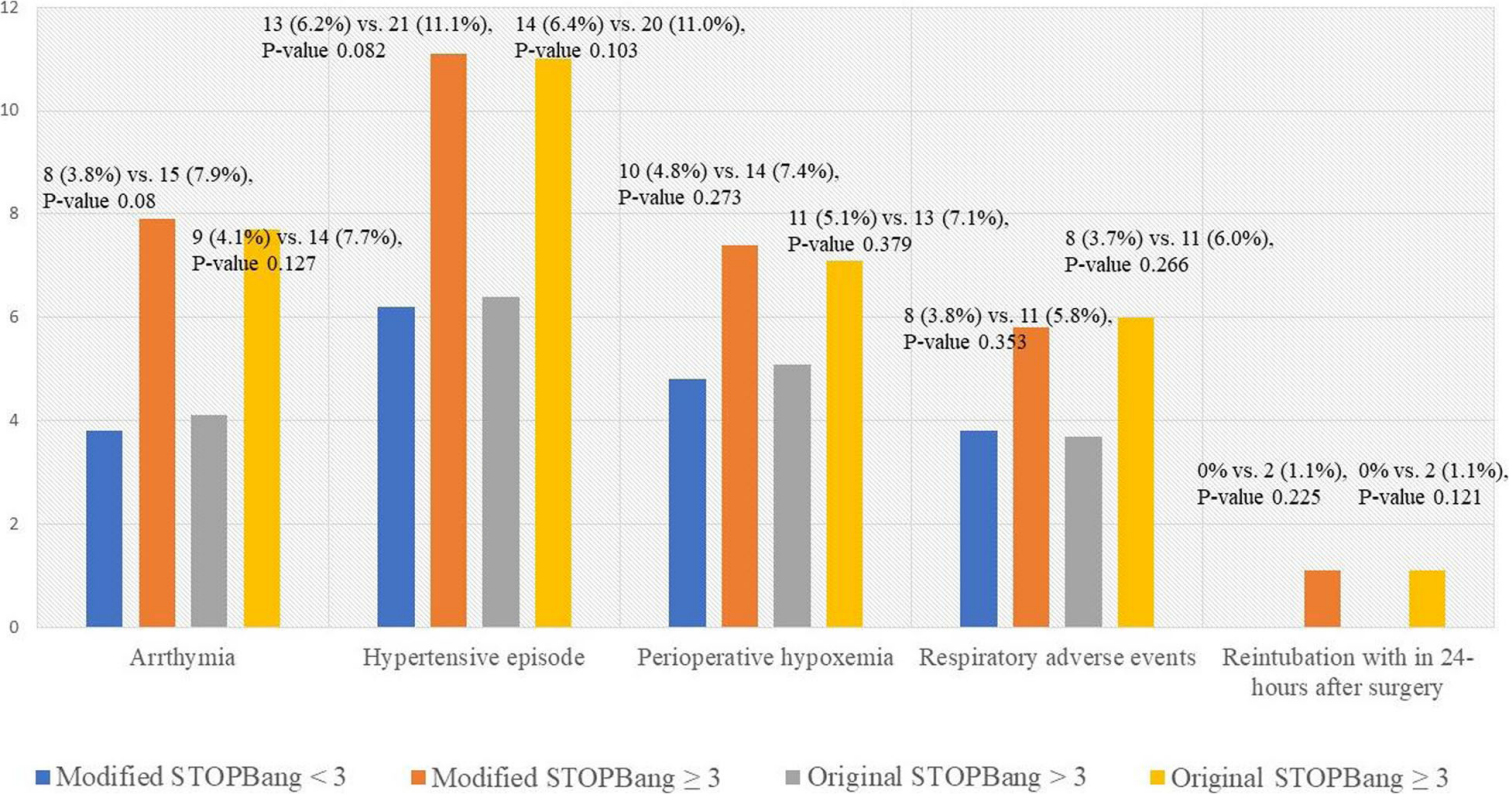
Perioperative operative adverse effects stratify risk by modified STOP-Bang questionnaire

Of the 400 patients, 73 experienced perioperative adverse events (18.3%). On the basis of modified STOP-Bang, the incidence of perioperative adverse events was 23.2% in patients with high risk for OSA and 13.8% in patients with low risk (*P*-value 0.016). While patient with high risk and low risk based on original STOP-Bang had incidence of perioperative adverse events at 22.5 and 14.7% (*P*-value 0.043). Figure 2 demonstrated the perioperative adverse events stratify risk by modified and original STOP-Bang. A logistic regression model was used to examine the incidence of perioperative adverse events while controlling for ASA physical status, type of anesthesia, preoperative oxygen saturation at room air, and total intraoperative opioid dosage (Table 2). Neither modified nor original STOP-Bang was associated with perioperative adverse events (adjusted OR 1.91 (95% CI 0.99–3.66), *P*-value 0.055) vs. 1.69 (95%CI, 0.89–3.21), *P*-value 0.106). In the subset analyses, modified STOP-Bang ≥3 was significantly associated with intraoperative adverse events, with an adjusted OR 2.52 (95% CI 1.18, 5.39, *P*-value 0.017). While original STOP-Bang was not statistical associated with intraoperative adverse events (adjusted OR 2.05 (95% CI 0.99, 4.29, *P*-value 0.055). Table 3 compares perioperative adverse events for the high-risk and low-risk OSA groups based on the modified and original STOP-Bang questionnaires. No significant difference was observed in the incidence of perioperative adverse events, as well as in modified or original STOP-Bang ≥5; adjusted OR 1.21 (95%CI 0.49, 3.02), *P*-value 0.678 vs. 1.13 (95%CI 0.43, 2.95), *P*-value 0.809.

**Table 2:** Analysis of risk factors for perioperative adverse events

	Univariate analysis		Multivariate analysis	
	OR, 95% CI	*P*-value	OR, 95% CI	*P*-value
Modified STOP-Bang ≥3	1.88 (1.12, 3.15)	0.017	1.91 (0.99, 3.66)	0.055
ASA physical status	1.93 (1.15, 3.25)	0.013	1.46 (0.77, 2.77)	0.245
Dyslipidemia	1.2 (0.66, 2.18)	0.544	–	–
History of stroke	4.63 (0.92, 23.41)	0.064	–	–
History of asthma	3.46 (0.76, 15.81)	0.109	–	–
History of renal disease	1.18 (0.52, 2.67)	0.700	–	–
General anesthesia	1.88 (1.04, 3.39)	0.035	1.4 (0.64, 3.08)	0.4
Preoperative oxygen saturation at room air (%)	0.79 (0.69, 0.91)	0.001	0.79 (0.68, 0.92)	0.002
Intraoperative opioid dosage (mg)	1.07 (1.03, 1.11)	0.001	1.06 (1.01, 1.11)	0.025

*Mg* milligram

**Table 3:** Compare odds ratio of based on perioperative adverse events based on modified STOP-Bang vs. original STOP-Bang questionnaire

Perioperative adverse events	Modified STOP-Bang ≥3		Original STOP-Bang ≥3	
	OR (95% CI), *P*-value	Adjusted OR (95% CI), *P*-value	OR (95% CI), *P*-value	Adjusted OR(95% CI), *P*-value
- Intraoperative	2.27 (1.24, 4.13), *P*-value 0.007	2.52 (1.18, 5.39), *P*-value 0.017	1.90 (1.06, 3.4), *P*-value 0.031	2.05 (0.99, 4.29), *P*-value 0.055
- PACU	1.71 (0.21, 3.91), *P*-value 0.201	1.15 (0.45, 2.96), *P*-value 0.771	1.87 (0.82, 4.27), *P*-value 0.138	1.27 (0.5, 3.27), *P*-value 0.614
- 24-h postoperative	0.83 (0.18, 3.74), *P*-value 0.804	0.76 (0.14, 4.02), *P*-value 0.743	0.90 (0.2, 4.06), *P*-value 0.887	0.79 (0.15, 4.19), *P*-value 0.781
- Overall perioperative adverse events	1.88 (1.12, 3.15), *P*-value 0.017	1.91 (0.99, 3.66), *P*-value 0.055	1.69 (1.01, 2.82), *P*-value 0.044	1.69 (0.89, 3.21), *P*-value 0.106

*CI* Confidence interval, *OR* Odds ratio, *PACU* Post-anesthetic care unit

Using ROC analysis, the modified STOP-Bang had a comparable AUC with the original STOP-Bang (AUC 58 (95% CI: 52–64) vs. 57 (95% CI: 50–63), *P* = 0.78). The sensitivity analysis indicated that BMI cutoffs of 25, 27, 27.5, 30, or 35 kg/m^2^ did not significantly improve the AUC (Table 4).

**Table 4:** Predictive performance of STOP-Bang screening tools at difference BMI cutoff against perioperative complications

BMI cutoff	Sensitivity	Specificity	PPV	NPV	AUC	*P*-value
Modified BMI 25 kg/m^2^	69.9 (58.0, 80.1)	48.9 (43.4, 54.5)	23.4 (17.9, 29.6)	87.9 (82.3, 92.3)	0.59 (0.53, 0.65)	0.340
Modified BMI 27 kg/m^2^	67.1 (55.1, 77.7)	53.5 (47.9, 59.0)	24.4 (18.6, 30.9)	87.9 (82.6, 92.1)	0.60 (0.54, 0.66)	
Modified BMI 27.5 kg/m^2^	63.0 (50.9, 74.0)	54.4 (48.9, 59.9)	23.6 (17.8, 30.2)	86.8 (81.4, 91.1)	0.59 (0.53, 0.65)	
Modified BMI 30 kg/m^2^	60.3 (48.1, 71.5)	55.4 (49.8, 60.8)	23.2 (17.4, 29.8)	86.2 (80.8, 90.6)	0.58 (0.52, 0.62)	
Original BMI 35 kg/m^2^	56.2 (44.1, 67.8)	56.9 (51.3, 67.8)	22.5 (16.7, 29.3)	85.3 (79.9, 89.7)	0.57 (0.50, 0.63)	

*AUC* Area under ROC curve, *BMI* Body mass index, *PPV* Positive predictive value, *NPV* Negative predictive value, *ROC* Receiver operating characteristics curve statistics

During the intraoperative period, both modified and original STOP-Bang can predict a greater incidence of difficult ventilation and Cormack–Lehane classification of laryngoscopic view ≥3 (Table 5). Moreover, high-risk OSA patients also had greater incidence for oxygen supplement during recovery period. No patient experienced myocardial infarction or congestive heart failure in this study.

**Table 5:** Intraoperative and postoperative adverse events stratify by modified and original STOP-Bang questionnaire

	Modified STOP-Bang		P-value	Original STOP-Bang		*P*-value
	Low (*n* = 210)	High (*n* = 190)		Low (*n* = 218)	High (*n* = 182)	
Difficult ventilation	11 (6.4%)	34 (24.6%)	< 0.001	12 (6.8)	33 (24.8)	< 0.001
Difficult intubation	2 (1.2%)	5 (3.5%)	0.252	3 (1.7%)	4 (2.9%)	0.475
Cormack–Lehane classification of laryngoscopic view ≥3	4 (1.9%)	5 (2.6%)	0.04	4 (1.8%)	5 (2.7%)	0.032
Need for oxygen supplement during recovery period	17 (8.1%)	42 (22.1%)	< 0.001	20 (9.2%)	39 (21.4%)	0.001

The incidence of postoperative ICU admission was greater in the high-risk group (7.9% vs. 2.4%, *P* <  0.012). However, the 30-day mortality and length of hospital stay were not significantly different between the two groups.

## Discussion

This study explored the association of modified STOP-Bang and perioperative adverse events in a Thai population. We found that the modified and original STOP-Bang score ≥ 3 or ≥ 5 were not significantly different in predicting the incidence of perioperative adverse events. However, modified STOP-Bang was associated with intraoperative adverse events as well as difficult ventilation, Cormack–Lehane classification of laryngoscopic view ≥3, the need for oxygen therapy during discharge from the postanesthetic care unit and intensive care unit admission.

Because of the disadvantages of the polysomnogram in terms of cost and availability, various screening tools for OSA have been adopted to identify patient risk. Among them, the STOP-Bang questionnaire displayed higher precision and was quicker and easier to use than the Berlin questionnaire or ASA checklist [6].

Screening for OSA using the STOP-Bang questionnaire identifies patients with an increased incidence of postoperative complications. In the retrospective cohort study of 5432 elective surgical patients [7], STOP-Bang ≥3 had greater incidence of intraoperative and early postoperative adverse outcomes (27% vs. 5.5%). Corso et al. [16] conducted a prospective study of 3452 elective surgery patients and found that patients categorized as “high risk” based on the STOP-Bang questionnaire ≥5 had a greater incidence of postoperative complications (9%) than low-risk patients (2%). A meta-analysis study also demonstrated that STOP-Bang high-risk patients had four times higher postoperative complications than low-risk patients [8]. Chan et al. conducted a multicenter, prospective cohort study in Chinese and Malaysian ethnicity patients to determine the association of high-risk STOP-BANG and 30-day risk of cardiovascular complications in patients who had a high risk for postoperative cardiovascular events. The results showed that being STOP-Bang ≥3 and ≥ 5 increased rates of ICU readmission, a composite endpoint of cardiac complications, and stroke [17]. However, our data did not demonstrate an association between STOP-Bang and perioperative adverse events. This may be because our cohort mainly comprised the general surgical population, which have fewer comorbidities than the patients in Chan et al.’s study. Furthermore, our main population was predominantly female, which may have a lower OSA incidence.

Comparing performance between modified and original STOP-Bang, the modified version showed a borderline statistically significant (*P* = 0.055), and it had a strongly significant with intraoperative adverse events with adjusted OR 2.52. While original STOP-Bang was not statistically significant with adverse events at the whole time point. However, from the ROC and sensitivity analysis showed that the modified and original STOP-Bang had comparable performance (AUC: 0.58 (0.52–0.64) vs. 0.57 (0.50–0.63), *P* = 0.78). From these findings may emphasize the results by Tan et al., who evaluate the validity of STOP-Bang at BMI cutoffs 35, 30, and 27.5 kg/m^2^ in an Asian population. The results revealed that a cutoff of BMI > 35 kg/m^2^ can be used in the Asian population, as lower BMI cutoffs did not improve the questionnaire performance [18]. Thus, the modified STOP-Bang had a comparable performance as the original STOP-Bang, but it had a greater association with intraoperative complications.

In addition, a high risk of OSA assessed using modified STOP-Bang increased the risk of difficult facemask ventilation and higher laryngoscopic view, consistent with a previous study [19], as well as increased the need for oxygen supplement during the recovery period. Therefore, our findings may help clinicians be prepared for a potentially difficult airway (i.e., by having special airway equipment ready), use short-acting anesthetic agents, and increase the use of regional anesthesia techniques in these patients.

The major strength of this study is its prospective design. One study limitation was that the operators were not blinded to the results of the STOP-Bang questionnaire, precluding the exclusion of bias in their clinical management. Furthermore, our patient cohort had a low incidence of cardiorespiratory complications, such as myocardial infarction and congestive heart failure. Therefore, the lack of association of STOP-Bang ≥3 with cardiopulmonary complications cannot be definitively stated because the results may be underpowered to identify intergroup differences. Finally, most of our study patients were women compared with other studies on OSA, which may be another confounder affecting the association between STOP-Bang and perioperative adverse outcomes.

In conclusion, this study demonstrated that patients at high risk of OSA, as determined using the modified or original STOP-Bang questionnaire, increased incidence of perioperative adverse events. However, both questionnaires did not statistically associate with perioperative complications. The modified and original STOP-Bang questionnaire can predict the risk of intraoperative adverse events, difficult ventilation, Cormack–Lehane classification of laryngoscopic view ≥3, oxygen supplementation during the recovery period, and ICU admission. Thus, it can be used as a triage tool to assist clinicians in implementing appropriate perioperative strategies for patients at high risk of OSA.

## Supplementary Information

**Additional file 1.**

## Data Availability

The datasets during and/or analyzed during the current study available from the corresponding author on reasonable request.

## Abbreviations

AHI: Apnea hypopnea index
ASA score: American Society of Anesthesiologists’ score
AUC: Area under the curve
BMI: Body mass index
CI: Confidence interval
H: Hour
ICU: Intensive care unit
IQR: Interquartile range
OSA: Obstructive sleep apnea
PACU: Post anesthetic care unit
RR: Relative risk
ROC: Receiver operating characteristics
SD: Standard deviation
