# Supplementary material for: Modified STOP-Bang for predicting perioperative adverse events in the Thai population
Source: BMC Anesthesiol. 2021 Apr 27;21:132. doi: 10.1186/s12871-021-01347-0 (PMC8077766; doi:10.1186/s12871-021-01347-0)
Supplement: Supplementary file 1 — Additional file 1. [file 12871_2021_1347_MOESM1_ESM.docx]

Appendix 1

| Definitions of Perioperative adverse events | |
| --- | --- |
| 1. Hypoxemia | Clinically significant oxygen desaturation to < 90% at room air requiring in management |
| 1. Arrythmia | New or worsening disturbance of heart rhythm requiring new treatment or a change in treatment |
| 1. Hypertension | Increase in systolic blood pressure to > 160, diastolic blood pressure > 110 mmHg or require anti-hypertensive treatment |
| 1. Myocardial infarction | New onset of chest pain, electrocardiogram change and increased cardiac enzyme during perioperative period |
| 1. Congestive heart failure | New onset of heart abnormalities that cause pulmonary edema |
| 1. Respiratory adverse events | New onset of bronchospasm requiring treatment or laryngospasm due to airway obstruction due to uncontrolled muscular contraction of laryngeal cords |
